# Causal Agent Investigation and Treatment of Dogs Diagnosed with Discospondylitis in a *Brucella canis* Endemic Region

**DOI:** 10.3390/vetsci11060279

**Published:** 2024-06-18

**Authors:** Eileen M. Donoghue, Sara D. Lawhon, Sharon C. Kerwin, Nick D. Jeffery

**Affiliations:** 1 Department of Small Animal Clinical Sciences, Texas A&M University, College Station, TX 77843, USA; donoghuedvm@exchange.tamu.edu (E.M.D.); skerwin@cvm.tamu.edu (S.C.K.); 2 Department of Veterinary Pathobiology, Texas A&M University, College Station, TX 77843, USA; slawhon@cvm.tamu.edu

**Keywords:** *Brucella*, *Aspergillus*, canine, blood culture, galactomannan

## Abstract

**Simple Summary:**

There are many causes of discospondylitis (infection in the discs and neighboring bones in the vertebral column), and it can be difficult to identify the relevant infection in all cases. In this study, we report the diseases we detected after recommending a specific series of tests to owners of affected dogs in this *Brucella canis* endemic region of the world. We found a moderate proportion (~12%) of cases that had *Brucella canis* infection (which is infectious to people) and suspected fungal infection (which can be very difficult to treat). Blood cultures were frequently (39%) positive for an infectious agent. The outcome for most cases, even those with suspected fungal disease, was good. It appears that blood culture is a helpful diagnostic method, and stabilizing implants can be safely used even when there is bone infection. Our results suggest that both fungal and *Brucella* spp. infections may be moderately common causes of discospondylitis in dogs in the USA, where *Brucella canis* is endemic.

**Abstract:**

Discospondylitis is a well-recognized disease in dogs, but the relative prevalence of causal infectious agents and efficiency of relevant diagnostic tests are not well-established. Medical record review identified 117 dogs diagnosed with discospondylitis in our clinic over a 5-year period. In 32 dogs, discospondylitis was diagnosed as an incidental imaging finding; 24 of these dogs had concomitant neoplasia. A likely causal infection was identified in 45 of the remaining 85 dogs in which blood and urine cultures, serology for *Brucella* spp., and galactomannan fungal antigen testing were recommended. Ten dogs were diagnosed with *Brucella canis*, and ten were diagnosed with suspected fungal infection. Brucella suis serology was negative in all 35 dogs that were tested. Blood cultures were positive in 28 of 71 (39%) tested dogs, and urine culture was positive in 12 of 79 (15%). Cultures were positive from the lesion site of four of eight dogs that underwent surgery and one of the five dogs that underwent image-guided lesion sample collection. Subluxation secondary to discospondylitis was stabilized with metallic implants in four dogs. A similar proportion of known satisfactory treatment outcomes at last follow-up were recorded in dogs that had suspected fungal disease, other bacterial infections, or were *Brucella*-positive and in those dogs with imaging diagnosis only, although some individuals continued to receive anti-microbial agents or showed recurrent signs. These data support the value of blood culture in discospondylitis and suggest a relatively high prevalence of infection with *Brucella* spp. and suspected fungal infection.

## 1. Introduction

Discospondylitis is a well-known cause of spinal pain and, sometimes, neurologic deficits in dogs [1]. It is caused by infection, usually bacterial but sometimes fungal, that commences in the vertebral endplates and progresses to involve the bodies of the vertebrae and the discs themselves. Most cases are thought to result from blood-borne infection settling in the vertebral endplates (in which the arteries make a hairpin bend and blood flow slows, allowing infectious organisms to adhere to the endothelium and subsequently penetrate the capillary walls) [2,3,4]. All ages of dog are susceptible, and pediatric animals can be affected through umbilical infections, although this route is more commonly incriminated in farm animals [5]. In older animals, the source of infection is often not apparent, although, in some cases, it has been linked to urinary tract or other pelvic infections [6,7,8]. 

Although it is a well-known disease, the prevalence of discospondylitis is relatively low [9], and most series include fewer than 50 cases. Clinical signs usually consist of pain, often manifest when the dog changes position, jumps, or otherwise places mechanical stress on the vertebral column. Typical reported cases often show acute onset and rapid progression, with rapid response to therapy, usually prolonged antibiotic administration [1]. Associated pain may be severe, and, if bone destruction progresses sufficiently, discospondylitis can lead to pathological vertebral fracture and spinal cord compression. However, other syndromes are recognized, in which dogs exhibit long-term pain without progression and poor response to therapy [2]. 

Definitive diagnosis of discospondylitis can be difficult, because it demands both diagnostic imaging features [4,10] and evidence of infectious agent in the intervertebral disc space and neighboring vertebral bodies. In practice, all these aspects are rarely identified in each individual, so the diagnosis is often presumptively based on imaging, sometimes radiographic features alone, such as bone proliferation and destruction that is centered on a disc space and involves both neighboring vertebrae [10,11]. Many such presumptively diagnosed reported cases are treated with broad-spectrum antibiotics without specific diagnosis of the infectious agent [12]. This approach is often successful, although some cases progress with development of pathologic fractures that require stabilization [13].

Over recent years, at our hospital, a perception developed that discospondylitis was commonly poorly responsive and recovery incomplete despite prolonged antibiotic therapy. Because of the known high prevalence of *Brucella canis* in dogs in this region of the USA [14] and the apparent association of this infection with chronic clinical signs (see [15]), we considered that this organism might be responsible for this specific presentation of discospondylitis. On the other hand, there are also previous data to suggest that *Brucella* spp. discospondylitis may not be especially common in our specific location [16]. Therefore, this report aims to summarize the results obtained during a 5-year period when a series of tests for a range of infectious diseases was recommended to owners of dogs diagnosed with discospondylitis in our clinic. 

## 2. Materials and Methods

To investigate causes of discospondylitis in more detail and to determine whether *B. canis* may be a prominent cause in this endemic region, a defined series of tests was recommended to owners of dogs with imaging signs consistent with discospondylitis that presented to the Neurology service at the Texas A&M University Small-Animal Hospital. The recommended diagnostic investigations (see below) consisted of cross-sectional imaging (magnetic resonance imaging [MRI], computed tomography [CT], or both), then blood sampling for antibodies to *B. canis* using the indirect fluorescent antibody (IFA) screening test and the agar gel immunodiffusion (AGID) test, serology for *B. suis* using the card agglutination test, urine culture, and blood culture. Blood culture results are reported below as the pooled result from the sampling protocol used for all cases: at least 3 samples, each taken from different blood vessels, temporally separated by an interval of at least 1 h and each collected into a separate 30 mL blood culture tube (BD Bactec Plus [BD 442023]) [17]. Ethical approval was not required for this study because it is a retrospective review of medical records concerning a naturally occurring disease in pet dogs presented to a veterinary clinic for diagnosis and treatment. 

At the end of the defined 5-year study period (1 January 2018–31 December 2022) we identified all possible discospondylitis cases diagnosed in our small-animal hospital by searching electronic medical records for ‘dog’, ‘discospondylitis’ and ‘diskospondylitis’. The full set of records was then hand-searched to remove cases in which the imaging was insufficient to make a reliable diagnosis. This decision was made individually by two investigators (NDJ and SCK) examining the records of all the questionable cases and determining whether they fulfilled the inclusion criteria described below; cases in which there was a difference of opinion were included or excluded through joint review and discussion. This method implies that definitive evidence of infection (such as positive blood culture) was not necessary for inclusion in this study. 

Images considered consistent with discospondylitis showed bone lysis and bone proliferation on both sides of at least one disc space [10,11,18] on radiographs, CT, or MR images. A positive imaging diagnosis of discospondylitis was made according to the following criteria: (a) on radiographs, erosion of the endplates visible on at least two views, plus concurrent bone proliferation [19,20]; (b) on CT images, erosion of portions of neighboring vertebral endplates plus proliferation or sclerosis of neighboring bone [10,11,15]; or (c) on MRI, lysis of neighboring vertebral endplates as apparent on T1-weighted (T1W) images or contrast enhancement of at least 2 of: nucleus pulposus, adjacent endplates, and neighboring soft tissue. T2-weighted (T2W) or short tau inversion recovery (STIR) hyperintensity or T1W contrast enhancement of endplates was not considered diagnostic *unless* accompanied by T2W or STIR hyperintensity or T1W contrast enhancement in neighboring soft tissue or accompanying bone erosion [10,11,18]. 

In many cases, bone lysis was visible as small ‘bubbles’ close to the endplate and with some similarity to Schmorl’s nodes [21]. Differentiation from degenerative endplate disease was predominantly through diagnosis of an infection, although, in this study, we considered cases to be suitable for inclusion if they had typical clinical and imaging signs and were then treated by the attending clinician with antibiotics. Regions of hyperintensity on T2W or STIR MR images within and, or, adjacent to intervertebral discs were not necessarily considered diagnostic of discospondylitis without confirmatory evidence from diagnosis of a systemic infection (blood, urine culture, or antibody testing) or CT images of bone lysis and proliferation because of the possible confusion with non-infectious lesions such as degenerative disc disease [21]. 

Diagnosis of infection was made through the following: (i) culture of blood, urine, or material collected during surgery or through image-guided sampling from lesions; (ii) serology for specific agents (*Brucella* spp.); or (iii) galactomannan antigen testing (for fungal infection, most commonly *Aspergillus* spp.). Diagnosis of fungal and *Brucella* spp. infections from antigen or antibody testing can be problematic because of the possibility of false-positive results. In this study, a diagnosis of fungal infection was made if there was a positive culture or the galactomannan concentration was either unequivocally high (i.e., >1.5) or, for marginally positive results, repeatedly above the threshold for positivity designated by the laboratory (i.e., 0.5). If the initial screening test (IFA) for *B. canis* was positive, it was followed up with an agar gel immunodiffusion (AGID) or tube agglutinin test (or positive blood or urine culture). Both initial and confirmatory tests needed to return a positive result for a dog to be considered ‘positive’ for *Brucella canis* [22]. Tests for evidence of *Brucella suis* infection (this region has a high population of feral hogs that are known to carry *B. suis* [23]) using serum in the card agglutination test (Texas A&M Veterinary Medical Diagnostic Laboratory) were also recommended to owners of all dogs irrespective of their home environment from mid-2020. Blood samples were also taken to identify galactomannan antigen (a polysaccharide constituent of fungal cell walls, primarily *Aspergillus* spp.; MiraVista Veterinary Diagnostics). In some cases, samples for culture were also acquired from the intervertebral disc or adjacent vertebral bone by CT-guided biopsy or during open surgery. 

Inoculated blood culture tubes were submitted to the Clinical Microbiology Laboratory (CML), where they were vented using a venting needle (Thermo Scientific™ Remel™ Venting Needle; Thermo Fisher Scientific, Waltham, MA, USA) and placed in an incubator set at 35° ± 2 °C with a 5% carbon dioxide environment (CO_2_ incubator). Anaerobic blood cultures were left unvented. Following incubation for 24 h, 48 h, and 7 days, a 0.1 mL aliquot of the inoculated blood culture medium was plated on agar plates, including trypticase soy agar supplemented with 5% sheep blood (blood agar plate; BD 221261), MacConkey agar (BD 221270), and Columbia CNA agar supplemented with 5% sheep blood (CNA plate; BD 221353). Blood agar and CNA agar plates were incubated in a CO_2_ incubator, while the MacConkey plates were incubated at 35° ± 2 °C with a room-air environment (air incubator). All plates were examined daily for bacterial growth. Anaerobic blood cultures were incubated in the same way as the aerobic cultures; however, the aliquot of medium was inoculated onto Brucella agar (BD) and CNA agar plates. These plates were incubated in an anaerobic environment created by placing the plates in anaerobic jars with an anaerobic gas pack (Mitsubishi Gas Chemical Company, Tokyo, Japan), with an anaerobic indicator (BD) to confirm that anaerobic conditions had been achieved. Plates were examined daily for anaerobic bacterial growth. 

Urine for culture was collected by cystocentesis. Urine culture was performed by plating 1 μL and 10 μL aliquots of urine onto separate blood agar plates, as well as swabbing a sample of the urine onto a MacConkey agar plate and then streaking the sample for isolation. The 1 μL and 10 μL blood agar plates were incubated at 35° ± 2 °C in a CO_2_ incubator, while the MacConkey agar plate was incubated in an air incubator. All plates were examined daily for three days for bacteria. Colony growth was quantified using the blood agar plates. Results were reported with the total number of colony-forming units (CFU) per mL in the urine specimen.

### Data Handling and Analysis

Details of all included cases were assembled in Excel spreadsheets to summarize the clinical signs, diagnostic features, location of lesions, infectious disease testing, treatment, and response to treatment. The cases were categorized according to the positive diagnostic tests in each individual as follows: (1) ‘*presumed etiologic agent*’ (i.e., consistent imaging *plus* positive infectious disease testing); (2) ‘*imaging diagnosis only*’ (imaging positive, infectious disease testing negative); or (3) ‘*incidental*’ (i.e., diagnosed on imaging carried out for reasons other than investigating spinal pain [such as searching for metastases in oncologic patients]). Duration of clinical signs was dichotomized into categories of less than or more than 6 weeks because: (i) owners were often not clear about how long clinical signs had been apparent; (ii) in some cases, it was difficult to distinguish whether clinical signs were related to discospondylitis or to concomitant diseases; and (iii) because clinical signs lasting 6 weeks or more would likely be associated with observable bone changes on imaging [24].

Summary statistics were calculated using Excel and included dog weight, age, gender, and neuter status. Many owners in this study did not know the exact age of their dogs, so we recorded ages to the nearest 0.5 year. To further explore the relative incidence of discospondylitis between German shepherds and Labradors (the breed most commonly presented to the Neurology Clinic), we extracted the number of each of these breeds admitted through the Neurology Clinic during the period of this study and calculated a risk ratio for definitive diagnosis of discospondylitis between these breeds, using Stata 17 (StataCorp, College Station, TX, USA). 

## 3. Results

Medical record search of the 5-year study period identified 117 dogs with imaging characteristics consistent with our criteria for inclusion. Of these, 85 dogs were presented to the Neurology service, and their owners were recommended the series of diagnostic investigations. In the remaining 32 dogs, the diagnosis of discospondylitis was *incidental* (i.e., detected during imaging for reasons other than spinal pain) (Table 1). Of the 85 dogs that underwent the specific series of diagnostic tests, 45 were diagnosed with a presumed causal infection and so allocated to the *presumed etiologic agent* category; the remaining 40 dogs were allocated to the *imaging diagnosis only* category because evidence of a causal infection was not detected. 

### 3.1. Cases with a Presumed Etiologic Agent Diagnosis (n = 45, Table 2)

Dogs in which an infectious causal agent had been identified had a mean age of 5.4 years (SD = 3.5), a mean weight of 29.6 kg (SD = 14.1) and consisted of many different types and breeds, although only six (13%) weighed less than 10 kg. The most commonly affected breeds were German shepherd (n = 11) and Labrador (n = 6). Of the total 45 dogs in this category, 30 underwent CT, 17 underwent MRI, and 8 were imaged using both modalities. A total of 25 of the 45 (56%) dogs had signs of discospondylitis at more than one site in the vertebral column; overall, the most common site was the L7/S1 disc, which was affected in 21/45 (47%) cases. In 20 dogs, the clinical signs had been noticed for longer than 6 weeks. 

**Table 2:** Tests performed on the 45 dogs in the ‘*presumed etiologic agent*’ group (in which an infectious cause was identified).

Test	Number Tested	Positive
Blood culture	37	28
Urine culture	39	12
Lesion culture	6	4
*Brucella canis* serology	39	10
*Brucella suis* serology	18	0
Galactomannan antigen	34	9 *

* One case diagnosed by blood culture.

**Figure 1:**
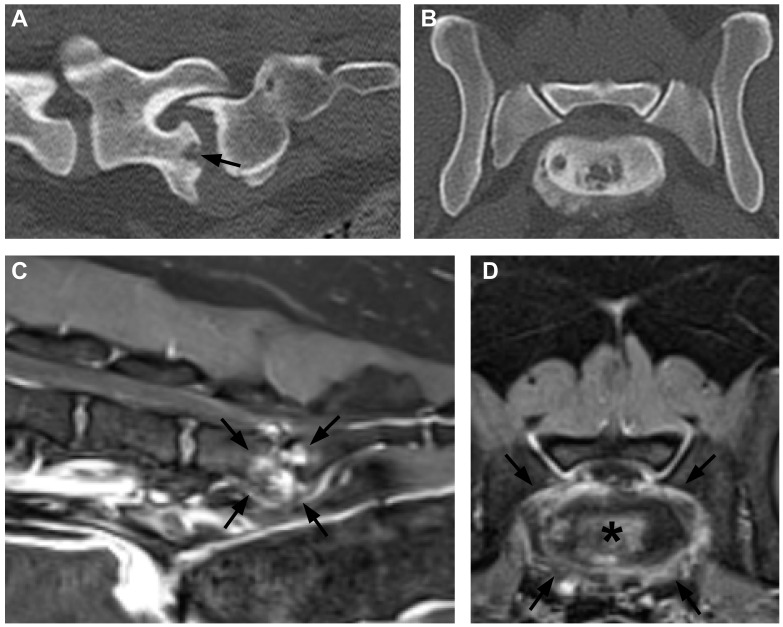
CT and MR images of discospondylitis in a dog in which *Brucella canis* infection was diagnosed. (**A**) Parasagittal CT at the lumbosacral junction, showing a ‘flask-shaped’ erosion of the L7 vertebral body (arrow) and (**B**) transverse CT through the caudal aspect of L7 vertebra. Both images show bone loss with adjacent sclerosis (plus ventral spondylosis). (**C**) Mid-sagittal and (**D**) transverse T1-weighted plus contrast MR images at the same location. There is widespread contrast enhancement in (*) and around (arrows) the intervertebral disc.

Of 37 dogs that had undergone blood cultures, 28 (76%) were positive (Table 1). Some dogs did not have blood culture performed because they had positive urine culture (n = 1), positive serology for *Brucella canis* (n = 3),or had undergone lesion sampling that detected an infectious cause (n = 4). Urine culture provided positive results in 12 of 39 tested [30%] dogs. Serology for antibodies to *B.canis* was submitted in 39 of these 45 dogs, and positive diagnosis of *B. canis* infection (i.e., positive culture, confirmatory positive testing after initial positive IFA test, or both) was made in 10, most commonly (7/10) from blood culture. A further seven dogs, which are included in the ‘*imaging diagnosis only*’ group below, showed a positive IFA test that was not supported by a positive confirmatory test. None of the dogs (n = 18) tested for *B. suis*/*abortus* were positive. In each of the *Brucella canis*-infected dogs, lesions on CT images were often small and smooth-edged, with a ‘flask’ shape of lysis surrounded by a thin rim of sclerotic bone (Figure 1).

A wide range of other bacteria were cultured from blood, urine, or other sites, of which the most frequent were *E.coli* (n = 7), *Staphylococcus pseudintermedius* (n = 6), and *Streptococcus canis* (n = 5). Ten dogs were diagnosed with suspected fungal infections. In nine of these dogs, the diagnosis was made by antigen detection (from 34 total submitted tests in this group, 32 serum, 2 urine, and 2 in which both urine and serum were tested) and one by culture from blood. Seven of these dogs were German shepherds (the remainder were a Golden Retriever, a mixed-breed dog, and a Labrador). 

**Figure 2:**
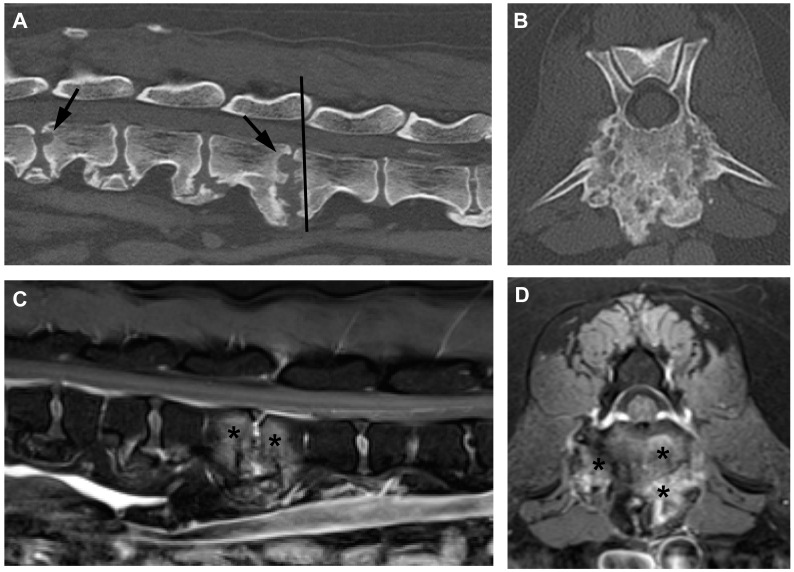
CT and MR images of discospondylitis in a dog in which fungal infection had been diagnosed by galactomannan antigen testing. (**A**) Mid-sagittal CT of the mid-lumbar region and (**B**) transverse CT through the cranial aspect of L4 indicated by line in (**A**). Note the multifocal lesions (arrows in **A**) and combined bone proliferation and lysis in (**B**). (**C**) Mid-sagittal and (**D**) transverse T1-weighted plus contrast MR images corresponding to the CT images. There is extensive contrast enhancement (*) in the vertebral bodies of L3 and L4 (in **C**) and within the vertebral endplate (in **D**).

Dogs with a diagnosis of suspected fungal discospondylitis tended to show larger areas of bone loss on CT, that sometimes included ragged, rough-edged regions of bone loss with less obvious neighboring sclerosis but with more pronounced neighboring proliferative bone (Figure 2).

**Figure 3:**
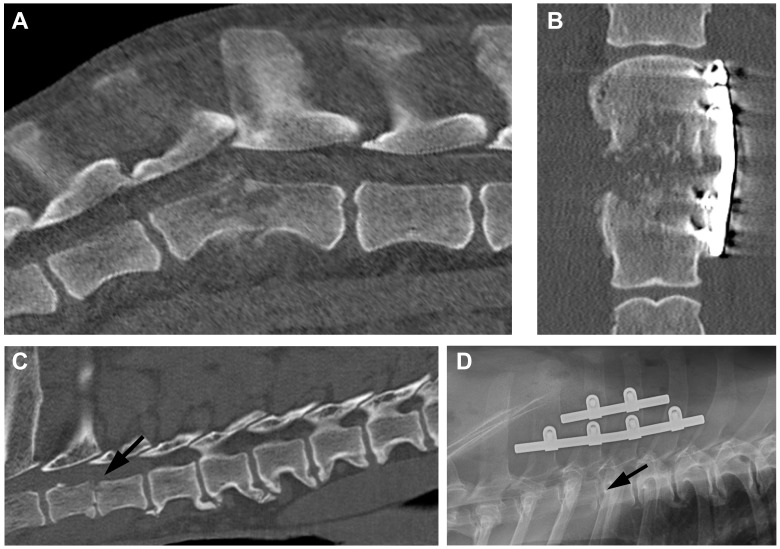
Surgically stabilized discospondylitis associated with infection with bacteria other than *Brucella* spp. (**A**): mid-sagittal CT image showing subluxation at L2/L3 associated with *Pseudomonas* discospondylitis in a Great Dane. (**B**): the lesion was stabilized with a 6-hole 3.5 mm DCP (visible in this dorsal plane CT image) plus a dorsally placed combined pin-and-wire tension band [25]. (**C**): mid-sagittal CT image showing T3/4 subluxation (arrow) associated with *S. canis* discospondylitis. (**D**): in this relatively stable region of the vertebral column the lesion was stabilized using a stacked De Puy CRIF implant [26] with 5 mm clamps and 3.5 mm screws. The stabilized site is indicated by an arrow.

Dogs diagnosed with bacterial infections other than *Brucella canis* showed a wide variety of lesion appearance (Figure 3); some dogs showed large destructive lesions and some showed more muted erosions with less apparent proliferation and raggedness. Overall, the type of infection was not invariably associated with a specific type of imaging appearance.

Dogs diagnosed with *B. canis* were treated with doxycycline and enrofloxacin or with cephalexin, and those with other bacterial infections were treated with antibiotics based on in vitro sensitivity testing. Dogs diagnosed with suspected fungal discospondylitis were treated with a variety of medications, including itraconazole, voriconazole, and terbinafine, along with antibiotics. One dog with repeatedly positive galactomannan antigen tests was treated with cephalexin alone (because of attending clinician doubt about the diagnosis). While there appeared to be no specific pattern to associate *Brucella canis* infections with either long or short duration of clinical signs at presentation (4/10 [40%] dogs with *Brucella canis* had signs for longer than 6 weeks), fungal infections trended toward a longer duration of clinical signs (8/10 [80%] showed clinical signs for longer than 6 weeks). In contrast, dogs with other bacterial infections (when not concomitant with fungal or *Brucella canis* infections) tended to exhibit a shorter duration of clinical signs (18/25 showed clinical signs for less than 6 weeks versus 7 [28%] dogs with a longer duration). 

At the time of data collection, eight (of the ten total) dogs diagnosed with *B. canis* infection were still alive at follow-up periods between one and twenty-two months after diagnosis but were all continuing antibiotic and analgesic (non-steroidal anti-inflammatory drugs [NSAIDs]) medications to alleviate clinical signs. One *Brucella canis*-infected dog was euthanized because of concerns for zoonotic risk to the owners. The dogs infected with other bacteria had variable outcomes. Twenty-two dogs were treated with antibiotics for longer than three months; nineteen were progressing satisfactorily between one and thirty weeks after diagnosis, when they were each lost to further follow-up. Of those, four dogs showed recurrence of spinal pain signs later (between 20 and 30 weeks after the initial diagnosis), but the cause of these signs was unknown. Two dogs showed signs of persistent pain at short-term follow-up (1 and 2 weeks) and were treated by the local veterinarian without further follow-up information. Four dogs were euthanized between 2 and 82 weeks after diagnosis because of intercurrent diseases (mostly neoplastic). Of the ten dogs affected with suspected fungal disease, one was euthanized at diagnosis, one died at home 16 days after imaging, and the remaining dogs were alive at follow-ups between 1 month and 4 years (Supplementary Material). One dog with a positive galactomannan antigen test progressed well, with reduction in pain and improvement in mobility for at least 4 months (last follow-up) following treatment with cephalexin alone.

Six dogs in this group underwent a surgical procedure for intervertebral stabilization (n = 3), lesion sampling (n = 2), or retrieval of a broken drill bit (associated with previous pelvic surgery at the referring clinic) from the infected intervertebral disc (n = 1), and culture samples were obtained from all these cases, of which four were positive for bacterial growth. Two dogs that underwent stabilization surgery were progressing well (ambulating without difficulty and well-controlled pain) when examined at the last available follow-up, 3 and 4 months post-operatively. One dog that was progressing well with a stabilized discospondylitis lesion at L3/4 was diagnosed with multicentric lymphoma at 4 weeks and was subsequently lost to follow-up. Three dogs underwent CT-guided needle aspiration of the lesion, one of which was diagnosed with an infectious agent (*S. pseudintermedius*). 

### 3.2. Comparative Breed Prevalence

During the period of data collection, there were 576 Labrador retrievers admitted through the Neurology service and 128 German shepherds. Within these cohorts, six Labradors and eleven German shepherds were definitively diagnosed with discospondylitis, producing a risk ratio for the diagnosis of discospondylitis in German shepherds (versus Labrador retrievers) of 7.7 (95%CI: 2.9–20.4). As noted above, the total of ten dogs diagnosed with suspected fungal infections included seven German shepherds and one Labrador retriever. Analysis of the prevalence of suspected causal agents between the two breeds revealed a risk ratio of 3.5 (95%CI: 0.96–12.9) for suspected fungal discospondylitis in German shepherds. 

### 3.3. Cases with an Imaging Diagnosis Only (n = 40)

Forty dogs were presumptively diagnosed with discospondylitis; in these dogs, imaging strongly supported the diagnosis but an infectious agent was not identified. The mean age of this group was 8.0 years (SD = 3.6), mean weight 23.8 kg (SD = 12.1), and, similar to the group with a *presumed etiologic agent* diagnosis, comprised a variety of breeds. The most common were Labrador retriever (n = 8) German shepherd (n = 4), and mixed-breed dogs (n = 3). Eight dogs (18%) weighed less than 10 kg. Twenty-one (53%) dogs showed clinical signs suggestive of discospondylitis for more than 6 weeks.

Twenty-nine of the dogs underwent CT and fifteen MRI scanning. Multifocal lesions were detected in 23 dogs (58%), and the L7-S1 disc space was affected in 18 dogs (45%). Imaging lesions were similar to those found in the *presumed etiologic agent* group, with some (n = 12) dogs showing collapse of disc spaces, intervertebral subluxation, or both, while the remainder showed a variety of sclerotic and lytic lesions characteristic of less aggressive discospondylitis. 

This group underwent recommended systematic testing for infectious diseases in the same way as the *presumed etiologic agent* group, but all the tests were negative (as necessary for inclusion in this group, Table 3). In all, there were 33 blood cultures, 36 urine cultures, and 33 dogs underwent serology testing for *B.canis*. Five dogs in this group showed a positive result on the initial IFA test for *Brucella canis* but were then negative upon subsequent confirmatory testing, and two dogs showed an initial positive IFA test but did not have follow-up confirmatory testing. Seventeen of the forty dogs in this category underwent serology for *B. suis*, and all were negative. Twenty-six dogs underwent galactomannan antigen testing (twenty-five from serum, one from urine only, and, in one dog, both were tested) and were all considered negative; two dogs showed weakly positive tests that were not repeatably higher than the threshold for declaring positivity (i.e., 0.5; MiraVista Diagnostics). Twelve dogs in this group were diagnosed with other diseases (excluding orthopedic disease) either at presentation or within a period of up to 8 months after diagnosis of discospondylitis.

Two dogs underwent image-guided lesion site sampling, which yielded no growth on culture. Two dogs underwent surgery: an 8.5-year-old neutered male Rottweiler and a 10-month-old Labrador retriever. In the Rottweiler, the cord was decompressed (soft tissue accumulation, presumably associated with discospondylitis, was removed from the epidural space) at T6-7. In the Labrador retriever, there was subluxation associated with the discospondylitis lesion, and the site was stabilized using an external fixator construct placed internally. In both cases, culture from the material retrieved from the surgical site was negative for growth, but, following 3 months of treatment with cephalexin, the dogs both recovered well from their presenting clinical signs. The remaining dogs were treated with various antibiotics (the great majority with cephalexin) and analgesics (NSAID and amantadine), but no dogs received antifungal medications. Twenty-six dogs were treated with antibiotics for longer than 3 months. Of the 40 dogs in this group, 12 were lost to follow-up within a 3-month period, so treatment effectiveness was difficult to ascertain, while 16 dogs recovered from their presenting signs and discontinued antibiotics after periods of 4–9 months. Eight dogs did not recover within a period of 5–9 months treatment with antibiotics, and one did not recover after 3 months of treatment. Three dogs relapsed, with recurrent signs of pain at 6, 10, and 11 months after an initial period of 3 months of antibiotics. 

### 3.4. Incidentally Diagnosed Discospondylitis (n = 32)

Thirty-two dogs were incidentally diagnosed with discospondylitis while undergoing routine diagnostic imaging for other reasons (predominantly cancer—see below). This group of dogs had a mean age of 11.6 years (SD = 2.7) and mean weight of 25.6 kg (SD = 12.8). Twenty-four dogs underwent CT imaging, and one underwent MRI (the remaining seven dogs were diagnosed from plain radiographs alone). Twenty-one of the thirty-two cases (67%) had multiple lesion sites: eighteen dogs had lesions affecting the mid-to-caudal thoracic lesion, whereas L7/S1 was affected in seven dogs. On the whole, discospondylitis lesions in this group of dogs were relatively subtle, generally consisting of smooth, rounded areas of bone lucency surrounded by dense regions of sclerotic bone. 

**Figure 4:**
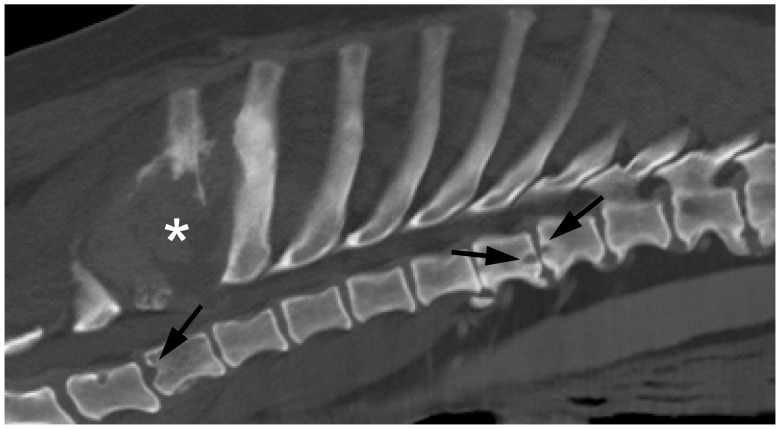
Multifocal discospondylitis (arrows) diagnosed incidentally in a dog undergoing CT imaging for a neoplastic spinal lesion (*). A specific causal agent was not diagnosed in this case.

The most common breeds in this group included Labrador retrievers (n = 5) and mixed-breed dogs (n = 4). Twenty-four dogs (77%) had neoplastic lesions (e.g., see Figure 4), and two dogs had an obvious potential source of bacterial infection (surgical site infection and septic peritonitis). Five dogs had diseases that were not specifically classified, but some of which could include possible sources of infection: stage-C heart valve disease, acute kidney failure, pituitary mass, implant for tibial plateau-leveling osteotomy, and prostatomegaly. The remaining dog had discospondylitis diagnosed at T5/6, concomitant with an acute intervertebral disc herniation at T13/L1, and did not receive any specific therapy for the suspect discospondylitis lesion. 

Infectious causes for discospondylitis were not exhaustively sought in this group (mainly because the intercurrent diseases were themselves very severe). Eleven dogs were tested for infectious disease in this group (including one blood culture, four urine cultures, eleven *Brucella canis* IFA tests, and nine galactomannan antigen tests [eight serum, one urine]), and all except one (in which *E. coli* was cultured from urine) were negative. Seven dogs were treated for discospondylitis (six with cephalexin and one with enrofloxacin). 

## 4. Discussion

During the 5-year study period, we identified 117 dogs presenting with diagnostic imaging characteristics consistent with discospondylitis, for which the owners of 85 dogs were recommended a systematic standardized investigation of etiology. Overall, our population characteristics were similar to those reported in a recent cohort of discospondylitis cases collected over a wide geographical area, although our cases tended to be slightly younger, and we identified a higher proportion of cases with lesions at L7/S1 [27]. A high proportion of cases (~55–65%) in each category showed multifocal lesions on imaging. In 45 dogs (53% of those whose owners had been recommended the series of diagnostic tests), i.e., the *presumed etiologic agent* group, we were able to identify a plausible infectious causal agent. *Brucella canis*, a potential zoonotic infection, was identified in 10 of these dogs, while 10 dogs were diagnosed with suspected fungal infections, which are uncommonly reported [28,29,30]. The remaining dogs were diagnosed with a variety of bacterial infections that have previously been described as causes of discospondylitis. Our *imaging diagnosis only* group showed similar demographics (dog size and breed) to the *presumed etiologic agent* group, although they tended to be slightly older (8.0 versus 5.4 years). The *imaging diagnosis only* group also exhibited similar imaging findings, albeit mostly with less marked bone lysis than the definitive group. In all, these findings suggest that the *imaging diagnosis only* group may likely have been infected with the same range of agents as the *presumed etiologic agent* group, but the agents simply eluded detection. This inference might perhaps be used to deduce how to treat cases in which an infectious agent has not been identified. 

Brucellosis as a cause of discospondylitis is of special concern because of the potential for zoonotic infection. Experimental infection of dogs with *B. canis* commonly does not persist, but, interestingly, those individuals in which the infection persists are not cured with antibiotic either [14,31], implying that dogs diagnosed with *Brucella* spp. must be considered as permanent potential sources of human infection. On the other hand, in this region of the USA, despite a known reasonably high prevalence of infection in dogs, laboratory-confirmed cases in people are uncommon [32], although recent serological evidence suggests that infection may be more common in people with occupational exposure to dogs (although still at low levels) than the rate of overt infections suggests [33]. *Brucella suis* is regarded as a higher-risk zoonotic disease, and it is known that it can be transmitted from dogs to humans (see commentary in [34]). 

The prevalence of *Brucella canis* infection in our population is difficult to compare with other recent reports because of possible differences in the time period in which they were accrued. Thus, Long et al. (2022) [15] reported on 33 cases of *Brucella canis* discospondylitis derived from four veterinary clinics in Arizona and Colorado, but it is not stated over what time period these were accumulated. Furthermore, our series only included a definite diagnosis of *Brucella* spp. infection if BOTH the initial IFA test AND another confirmatory test proved positive (OR a positive blood culture); if we included the cases in our *imaging diagnosis only* group that had a single initial positive test (n = 7), then our rate of positive diagnosis would be considerably higher. Although only 35 dogs were serologically tested for *B. suis*, all that were tested were negative, suggesting that the risk of infection with this pathogen (through exposure to wild hogs or their secretions) is low. The predominant risk of *Brucella* spp. infection therefore appears to be from contact with other dogs infected with *B. canis* or their infected material. Texas may be a region in which this risk is higher than some other locations, especially those within Europe, because there is no state-mandated euthanasia of infected cases, implying that the prevalence of the bacterium is higher than within areas with more stringent public health approaches. Nevertheless, there appears to be no discernible pattern regarding which dogs develop *Brucella canis* discospondylitis; similar to another recent report [15], any breed or age appears to be at risk.

Anecdotally, it is often considered that dogs with *Brucella canis* that are treated with antibiotics show relapse if the antibiotics are withdrawn, so, perhaps, episodes of bacteremia are less frequent during the period of antibiotic administration. On the other hand, there is the possibility of a confusion of effects, because most dogs are treated with analgesics together with antibiotics so the perceived response might derive from those rather than the antibiotics. There are strong arguments that antibiotic use for discospondylitis should not be unduly prolonged, both because of the unlikelihood of satisfactory resolution [14] and the hazards associated with indiscriminate antibiotic use and development of widespread resistance [35]. The information on *Brucella canis* infections in this case series suggests that this infectious agent may be responsible for the chronic and poorly responsive but almost non-progressive discospondylitis syndrome associated with multiple, small, smoothly marginated areas of vertebral endplate lysis that appears to be relatively common in this region of the USA. 

The *incidental* group also provided some interesting hints about the pathogenesis of discospondylitis in dogs. Dogs in this group were much older than the other groups (11.6 versus 8.1 and 5.4 years) and had lesions in different vertebral sites (predominantly thoracic versus L7). Although a small minority of this group had obvious distant sites of potential infection, the preponderance was of dogs with neoplastic lesions. This suggests another important potential etiological factor: immunosuppression. It is well-established that neoplasia, especially malignant types and, often, its treatment (i.e., chemotherapy), is associated with immunosuppression [36]. 

Ten of the forty-five dogs (22%) with a *presumed etiologic agent* diagnosis had a suspected fungal infection. Fungal discospondylitis in dogs has only been reported occasionally (e.g., [28,29,30]), but, anecdotally, it appears to be reasonably prevalent in this region. It is possible that the relatively high rate of fungal disease diagnosis may have also been because a large proportion (62/85 = 73%) of our cases underwent galactomannan antigen testing in comparison to other large-scale studies on discospondylitis [27]. This disease is strongly associated with German shepherd dogs, which have long been known to have a deficiency in cell-mediated immunity and a susceptibility to fungal infection [37]. German shepherd dogs are relatively uncommonly presented to our Neurology service, but they were disproportionately common amongst our suspected fungal disease diagnoses. In this series, it appears that suspected fungal discospondylitis may be a chronic infection that, in many cases, is satisfactorily responsive to antifungal medications such as voriconazole and terbinafine. Although this geographic region is thought to be one in which fungal disease is prevalent, it seems improbable that the specific climatic conditions would favor this infection because *Aspergillus* (the most commonly incriminated fungal pathogen in canine discospondylitis) is an organism with a more or less ubiquitous distribution [38], 0so it seems unlikely that dogs in this area would be more exposed than those attending other veterinary centers. 

In this series, some dogs showed persistent pain or recurrence of clinical signs consistent with discospondylitis even when treated with appropriate anti-microbial agents. This finding might suggest that surgical stabilization should be considered more often in discospondylitis in dogs to alleviate these problems, especially since it was associated with good outcomes in this series. Veterinarians could follow similar indications for surgery in this condition to those applied in humans [39]. Stabilization surgery can be beneficial in discospondylitis, as in long-bone osteomyelitis [40], because it restricts motion (thereby also alleviating nerve compression and pain), allowing blood vessels to traverse the damaged region and, therefore, allow bone healing and fusion. Because of the known increase in bacterial adhesion compared to metallic implants [41], there are clear reasons to avoid using polymethylmethacrylate (combined with screws or pins) as fixation when vertebrae are infected, although it has been successfully used for this purpose [42]. Metal implants also provide a substrate for bacterial attachment, but the good results obtained following their use in human spondylodiscitis imply that the robust stabilization they provide outweighs this disadvantage [38,43,44]. In our series, internal stabilization for discospondylitis in dogs appeared to be similarly effective. 

Diagnosis of discospondylitis raises some interesting questions, mainly because unequivocal diagnosis would require histologic examination of affected tissues (to detect inflammation and, possibly, infection), and it is plausible that evidence of inflammation at or adjacent to intervertebral discs may sometimes be non-infectious in origin (e.g., [45,46]), accounting for the inability to detect an infectious agent in all cases. Nevertheless, it would be more plausible to consider that most cases in which a pathogen is not detected arise through a lack of sensitivity in detection rather than its absence. Even so, imaging alone would be imprecise, because some animals have infection but inapparent bone changes (perhaps because of an infection of low pathogenicity or early in the course of disease) and some have clear bone changes unaccompanied by a diagnosed infection. Moreover, the diagnostic criteria may vary between individual image reviewers, and it is possible that different series of cases of discospondylitis in dogs use different inclusion criteria (especially when comparing older reports based on plain radiographic diagnoses versus more recent studies reliant upon MRI). Nowadays, MRI is widely considered to be the most diagnostic modality [10,47], although there is a risk of confusion with other diseases of the vertebral endplate [22]. In the current study, the two image reviewers had most disagreement regarding diagnosis on MR images (versus CT agreement), in particular, regarding the interpretation of changes in contrast enhancement adjacent to the endplates. Although contrast enhancement of these areas in MR images is clearly abnormal, it is non-specific and can be associated with degenerative changes [18,21]. In MRI, there is also low clarity in the imaging of the bone endplates, so it is difficult to reach consensus about the loss of bone density or proliferation, which are clearly defined using CT. While MRI can show changes in bone responses and even aids in delineating vasculature using contrast, it provides poor information on bone lesions, which are traditionally required for diagnosis. Therefore, in this study, bone changes on MRI without evidence of bone lysis and proliferation on any imaging modality were largely attributed to non-infectious causes when considering cases for inclusion in this series and corresponded to dogs having a long history of non-progressive clinical signs before presentation, in the absence of treatment with antibiotics. 

### Study Limitations

Although we recommended a specific set of diagnostic tests to owners, there was no funding available to support this investigation, so not all tests were carried out in every individual. Although this limits the conclusions that can be drawn regarding the diagnostic yield of each test, it enhances the generalizability of our results [48], because our data would be strongly indicative of the rate of positive diagnosis that might be expected if other veterinarians were to recommend a similar set of diagnostic tests in their clinics (because, in real life, as in our series, not every owner can afford to have every test performed on their dog). 

## 5. Conclusions

We conclude that our approach to identification of causal agents of discospondylitis in dogs, in which we recommended a series of diagnostic tests to owners, enabled us to detect *B. canis* and suspected fungal infections, both of which have major implications for subsequent case management, in a substantial minority of cases. Of a total of 71 dogs from which blood cultures had been obtained, 28 (39%) were positive; in comparison, only 12 (15%) of a total of 79 urine cultures were positive. Previous reports have promoted urine culture as a diagnostic method in discospondylitis and suggested that they be prioritized against blood cultures [49]. In contrast, while bearing in mind the caveats above, our data suggest that blood cultures should be prioritized. It appears that suspected fungal discospondylitis can be managed satisfactorily with antifungal medications for prolonged periods in many cases. Our results also suggest that, where feasible (i.e., where single vertebral unit stabilization might substantially reduce pain, notably, the lumbosacral junction), it might be beneficial to consider surgical stabilization in a greater proportion of cases.

## Figures and Tables

**Table 1:** Demographics of 117 dogs diagnosed with discospondylitis.

	Diagnostic Category					
	Presumed Etiologic Agent			Presumed Etiologic Agent	Imaging Diagnosis Only	Incidental
	Brucella spp.	Fungus	Other Bacteria			
Male castrated	6	5	10	21	18	18
Male intact	1	1	5	7	7	3
Female spayed	2	4	9	15	15	11
Female intact	1	0	1	2	0	0
Mean age (year)	3	5.5	6.3	5.4	8.0	11.6
Mean weight (kg)	29.3	29.8	29.5	29.6	23.8	25.6
CT	5	7	18	30	29	24
MRI	2	2	13	17	15	1

**Table 3:** Tests performed on the 40 dogs in the ‘imaging diagnosis only’ group (in which an infectious cause was not identified).

Test	Number Tested
Blood culture	33
Urine culture	36
Lesion culture	4
*Brucella canis* serology	33
*Brucella suis* serology	17
Galactomannan antigen	26

## Data Availability

All data are contained within the article.

## Supplementary Materials

The following supporting information can be downloaded at https://www.mdpi.com/article/10.3390/vetsci11060279/s1: Video S1: CT (video images) of thoracolumbar vertebral column of a 4-year-old neutered male German shepherd dog presenting with severe spinal pain and kyphosis and a diagnosis of presumed fungal discospondylitis. Video S2: Same dog after treatment for 4 months with voriconazole (4 mg/kg BID) and terbinafine (10 mg/kg BID).
