# Supplementary material for: Causal Agent Investigation and Treatment of Dogs Diagnosed with Discospondylitis in a Brucella canis Endemic Region
Source: Vet Sci. 2024 Jun 18;11(6):279. doi: 10.3390/vetsci11060279 (PMC11209358; doi:10.3390/vetsci11060279)
Supplement: Supplementary file 1 [file vetsci-11-00279-s001.zip › vetsci-2931652-supplementary final/Supplementary material legend.pdf]

Supplementary Material

Video S1: CT scan of the thoracolumbar vertebral column of a 4 year old neutered male German shepherd dog that presented with severe spinal pain and was diagnosed positive for galactomannan antigen.

Video S2: The same dog after 4 months' treatment with voriconazole and terbinafine.
